# *Cfdp1* Is Essential for Cardiac Development and Function

**DOI:** 10.3390/cells12151994

**Published:** 2023-08-03

**Authors:** Panagiota Giardoglou, Panos Deloukas, George Dedoussis, Dimitris Beis

**Affiliations:** 1 Zebrafish Disease Model Laboratory, Center for Clinical, Experimental Surgery & Translational Research, Biomedical Research Foundation, Academy of Athens, 11527 Athens, Greece; tota_giardoglou@yahoo.gr; 2 Department of Nutrition and Dietetics, School of Health Science and Education, Harokopio University of Athens, 17676 Athens, Greece; dedousi@hua.gr; 3 Clinical Pharmacology, William Harvey Research Institute, Barts and The London Medical School, Queen Mary University of London, London E1 4NS, UK; p.deloukas@qmul.ac.uk; 4 Laboratory of Biological Chemistry, Faculty of Medicine, School of Health Sciences, University of Ioannina, 45110 Ioannina, Greece

**Keywords:** zebrafish models of human disease, cardiovascular development, coronary artery disease, arrythmias, bradycardia, *cfdp1*

## Abstract

Cardiovascular diseases (CVDs) are the prevalent cause of mortality worldwide. A combination of environmental and genetic effectors modulates the risk of developing them. Thus, it is vital to identify candidate genes and elucidate their role in the manifestation of the disease. Large-scale human studies have revealed the implication of Craniofacial Development Protein 1 (CFDP1) in Coronary Artery Disease (CAD). CFDP1 belongs to the evolutionary conserved Bucentaur (BCNT) family, and to date, its function and mechanism of action in Cardiovascular Development are still unclear. We utilized zebrafish to investigate the role of *cfdp1* in the developing heart due to the high genomic homology, similarity in heart physiology, and ease of experimental manipulations. We showed that *cfdp1* was expressed during development, and we tested two morpholinos and generated a *cfdp1* mutant line. The *cfdp1^−/−^* embryos developed arrhythmic hearts and exhibited defective cardiac performance, which led to a lethal phenotype. Findings from both knockdown and knockout experiments showed that abrogation of *cfdp1* leads to downregulation of Wnt signaling in embryonic hearts during valve development but without affecting Notch activation in this process. The *cfdp1* zebrafish mutant line provides a valuable tool for unveiling the novel mechanism of regulating cardiac physiology and function. *cfdp1* is essential for cardiac development, a previously unreported phenotype most likely due to early lethality in mice. The detected phenotype of bradycardia and arrhythmias is an observation with potential clinical relevance for humans carrying heterozygous CFDP1 mutations and their risk of developing CAD.

## 1. Introduction

Multiple factors contribute to the severity of CVD traits and it still remains unclear to which extent genetics and environmental elements lead to the disease manifestation. The suggested predisposition of the CVD appearance is currently a primary focus of research interest. Genome-wide association studies have identified thousands of robust associations (genome-wide significance, *p* < 5 × 10^−8^) between disease traits and genetic loci. For coronary artery disease (CAD), 66 loci have been proposed to account for approximately 12% of CAD heritability [1,2,3,4]. Moreover, it has also been reported that a larger number of putative loci is found close to the false discovery rate (FDR) of 5%. Recently, a study that used UK Biobank data [5] to evaluate the validity of FDR loci and conducted a meta-analysis using CAD GWAS identified new loci at GWAS significance that were previously on 5% FDR, providing support that variants in this threshold could hold the key for a higher percentage of heritability [4]. Recent publications derived from the analysis of GWAS human data have highlighted the involvement of *BCNT/CFDP1* (Bucentaur/craniofacial development protein 1, hereafter referred to as *CFDP1*) in the determinants of risk factors for CAD, blood pressure, aortic diameter, neuroblastoma (NB) susceptibility and carotid intima–media thickness, raising the interest for a deeper understanding of the functional analysis of the *CFDP1* gene in cardiovascular development and function [6,7,8,9,10].

The human *CFDP1* is a protein-coding gene belonging to the evolutionary conserved Bucentaur superfamily, which is classified by the uncharacterized BCNT domain of 80 amino acids (aa) at the C-terminal region. It is located at the reverse strand of chromosome 16 and consists of seven exons, which encode for a protein product of 299 aa, and it is flanked by the *BCAR1* (breast cancer antiestrogen resistance 1) and *TMEM170A* (transmembrane protein 170A) genes. The BCNT protein family is widespread among the species, and their orthologues are also found in yeast *Saccharomyces cerevisiae* (SWC5), fruit fly *Drosophila melanogaster* (YETI), mouse *Mus musculus* (CP27) and zebrafish *Danio rerio* (CFDP1) [11]. The fact that BCNT is evolutionarily conserved highlights the important role of this superfamily, which was first detected in bovine brain extracts using monoclonal antibodies against a rat GTPase-activating protein with the same epitope [12]. Although the *CFDP1* gene is highly conserved, there is limited knowledge about its function, both at the cellular and organismal levels. The yeast *Swc5* gene is essential for the optimal function of the chromatin remodeler SWR, which has histone exchange activity in an ATP-dependent manner [12,13,14,15]. Studies in *Drosophila melanogaster* have shown that the loss of the BCNT gene *Yeti* causes lethality before pupation and that it mechanistically provides a chaperone-like activity that is required in higher-order chromatin organization by its interaction with histone variant H2A.V and chromatin remodeling machinery [16]. Recently, it was shown that the zebrafish *gazami* mutants carry a point mutation in the 3′ end of the *cfdp1* gene, resulting in a truncated protein [17]. It is proposed that *cfdp1* controls neural differentiation and the cell cycle in the cerebellum and retina. In the same context, another study focusing on the functional analysis of zebrafish *cfdp1* has shown evidence for its role in craniofacial structure and bone development [18]. Mammalian BCNT proteins have also been characterized as molecular epigenetic determinants via their association with chromatin-related proteins [19]. The mouse BCNT gene *cp27* was suggested to mediate early organogenesis and a high level of its expression was demonstrated in developing mouse teeth, heart, lungs, and liver [20,21,22]. In addition, the embryonic lethality prior to organogenesis of the *cp27* knockout mouse model, as reported in the International Mouse Phenotyping Consortium, (https://www.mousephenotype.org/data/genes/MGI:1344403 accessed on 1 March 2023) indicates the role of the gene during early embryogenesis. Due to insufficient tools to study the localization and function of *CFDP1*, in vivo studies remain relatively limited. The *cfdp1* locus in zebrafish was initially misassigned to the RLTPR (RGD motif, leucine rich repeats, tropomodulin domain and proline-rich containing) gene and the role of BCNT/CFDP1 in the heart has not been investigated as yet. Thus, while there are sparse studies assessing the role of *CFDP1,* mainly in the chromatin remodeling complex via the maintenance of chromosome organization [23,24], its role in heart development and physiology remains to be addressed and evaluated, especially since GWAS studies have revealed the association of CFDP1 with CAD in humans [25].

For this purpose, we utilized the zebrafish as a model organism, a valuable vertebrate tool to model human cardiovascular development and diseases [26,27,28]. The physiology of zebrafish development offers a precious advantage to study mutations that result in early embryonic lethality. For instance, mutations in the *cardiac troponin T (cTnT)*, also known as *sih* (*silent heart*) mutants, exhibit a non-contractile heart phenotype; the *sih* embryos survive up to 5 dpf (days post fertilization) as they uptake adequate oxygen through diffusion and are not dependent on a functional cardiovascular system and blood circulation until that developmental stage [29]. This ability allows for the characterization of mutations that are embryonically lethal to other vertebrate models.

In this work, we aimed to study the previously unappreciated role of *cfdp1* during the development of the embryonic heart in order to elucidate its involvement in proper cardiac function. We showed evidence that the cardiac expression of *cfdp1* is apparent during early developmental stages and plays an essential role in myocardial trabeculation. Due to *cfdp1* abrogation, embryos display heart dysfunction, including contractility impairment and the development of cardiac arrhythmias, supporting its role in proper cardiac performance. *cfdp1*^−/−^ mutants and a percentage of heterozygous do not survive to adulthood as their hearts develop severe arrhythmias and stop by 10 days post fertilization, suggesting a partially dominant phenotype of *cfdp1* loss of function. In addition, mutant *cfdp1* embryonic hearts exhibit downregulation of the Wnt signaling pathway in the mesenchymal cells of the inner valve region during valvulogenesis without affecting Notch activation in this process.

## 2. Materials and Methods

### 2.1. Fish Housing and Husbandry

Adult zebrafish were maintained and embryos were raised under standard laboratory conditions, at 28 °C at a day–night cycle according to the Recommended Guidelines for Zebrafish Husbandry Conditions [30]. The zebrafish transgenic reporter lines used in the study were Tg(*myl7:GFP*) for myocardium [31], Tg(*fli1:EGFP*) [32] for endothelial cells, Tg(*Tp1:mCherry*) for Notch-responsive cells [33] and Tg(7x*TCF-Xla.Siam:nlsmCherry*) [34] for Wnt-activated cells. CRISPR-induced mutations to study cardiovascular genes, genotyping and adult handling of animal experimentation protocols were approved from the Bioethics and Animal Committees of BRFAA and the Veterinary Department of Attica region (numbers 247,914 and 247,916, 08/04/20) for the facility EL 25 BIOexp 03. Embryos and larvae were anesthetized by adding 0.4 mL tricaine 0.4% (MS-222, Ethyl 3-aminobenzoate methanesulfonate salt) (Apollo Scientific, Manchester, UK cat.# BIA1347) in 25 mL E3 embryonic water at a final concentration of 0.0064% (*v*/*v*). Pigmentation of 24 hpf embryos was prevented by adding phenylthiourea (PTU, Sigma-Aldrich, St. Louis, MO, USA, P7629) in E3 embryonic water at a final concentration of 0.003%

### 2.2. gRNA and Cas9 mRNA Synthesis

The identification and design of target sites to specifically knock out the *cfdp1* gene was performed by using the online CRISPR design tool CHOPCHOP v2 (https://chopchop.cbu.uib.no/ accessed 1 October 2019). The selected target site is located in exon 3 and the sequence is 5′-CAGTAGGAGACATTGAAGAGCGG-3′ (where the downstream underscored 3nt CGG represents the PAM sequence, which is immediately adjacent to the target site without being part of it). CRISPR gRNA mutagenesis was designed according to [35] and, briefly, the protocol used is as follows: the oligos were synthesized, annealed and cloned in pT7-gRNA (Addgene, Watertown, NY, USA, plasmid #46759). For making nls-zCas9-nls mRNA, the DNA vector pT3TS-nCas9n (Addgene, Watertown, USA, plasmid #46757) was linearized by XbaI digestion and purified.

### 2.3. Microinjection in Zebrafish Embryos

Microinjections were performed into one-cell-stage zebrafish embryos. The final concentration of the injection mixture for the generation of the *cfdp1* mutant line was as follows: 300 ng/μL Cas9 mRNA, 50–100 ng/μL gRNA, 10% (*v*/*v*) Phenol Red, 20 mM HEPES and 120 mM KCL. *cfdp1* ATG-blocking and *cfdp1* splice morpholino were synthesized by GeneTools LLC, Philomath, OR, USA. The sequences are TCTGAATAATTCATTCTTGTGTCGT and CCAAATCGGGCAGCCCTACCTCATA, respectively. The final concentration of antisense *cfdp1* morpholino used was 0.4 mM MO. The primers used for RT-PCR in the *cfdp1*-splice-injected embryos are as follows: Fw: 5′-GCACGTACGACACAAGAATG-3 and Rev: 5′-GATCTGCTGTACTTGCCTCG-3′.

### 2.4. Tail Amputations and Genotyping

The target region of *cfdp1* was PCR amplified using flanking genomic primers: forward, 5-GGAGGCCTCAAACTGGTGGAG-3′, and reverse, 5-CTTCTGAGAGCTTGCACTTGG-3′. Amplicons were then prepared for Sanger sequence after product cleaning using ExoSAP (New England Biolabs, Ipswich, MA, USA #M0293S, #M0371S). Alternatively, amplicons were visualized on a 2% agarose gel after diagnostic digestion to confirm the loss of the SapI cutting site inside the target site.

### 2.5. RT-PCR

For reverse-transcription PCR, synthesized cDNA was the template for PCR amplification and the primers that were used in this study are listed as follows: *cfdp1*, forward: 5′-GAGACATTGAAGAGCGGCAG-3′, reverse: 5′-CGACTTCTCCAGAGTGCTCA-3′; *actin*-2*b*, forward: 5′-CGAGCTGTCTTCCCATCCA-3′, reverse: 5′-TCACCAACGTAGCTGTCTTTCTG-3′. Quantification was performed in ImageJ2 Software Version 1.53c and relative expression was normalized to *actin-2b* as a reference gene.

### 2.6. RNA Extraction and Quantitative Real-Time PCR

qPCR reactions were analyzed on a Roche Lightcycler 96 (Roche Life Science, Penzberg Germany) using KAPA SYBR FAST (Sigma-Aldrich, KK4611). Cycling conditions were as follows: 2 min at 50 °C and 10 min at 95 °C followed by two-step PCR for 40 cycles of 15 s at 95 °C and 60 s at 60 °C. The relative expression of *cfdp1* was normalized to the average of the stably expressed reference gene *act2b*, and both the ΔCts and 2^−ΔΔCt^ values were calculated and presented. The primers used are as follows: *actin2b:* Fw: 5′-CGAGCTGTCTTCCCATCCA-3′, Rev: 5′-TCACCAACGTAGCTGTCTTTCTG-3′; and *cfdp1*: Fw: 5′-GAGACATTGAAGAGCGGCAG-3′, Rev: 5′-CTTCTGAGAGCTTGCACTTGG-3′.

### 2.7. Whole-Mount In Situ Hybridization and Immunohistochemistry

Whole-mount RNA in situ hybridization (ISH) using the *cfdp1* antisense probe was performed in embryos, according to *The Zebrafish Book* [36]. Primers for the generation of *cfdp1* probes were forward, 5′-GAGACATTGAAGAGCGGCAG-3′, and reverse, 5′-CGACTTCTCCAGAGTGCTCA-3′, amplifying a 458 bp fragment from +314 to 771 from the ATG codon. Phalloidin-633 (1:300 in PBT) for filamentous actin staining was used.

### 2.8. Imaging

Zebrafish embryos were anesthetized with 0.006% (*v*/*v*) tricaine, placed dorsally on separate cavities of a glass slide (Marienfeld Superior, Lauda-Königshofen, Germany, 10622434), mounted on 1.2% low-melting agarose and a drop of E3 embryonic water was added on top of the semi-solidified mounting medium for the maintenance of humidity. For in vivo imaging, fluorescent and brightfield videos of 10 s were recorded by microscope inverted Leica DMIRE2, Wetzlar, Germany, with a mounted Hamamatsu ORCA-Flash4.0 camera. Confocal imaging was performed using a Leica TCS SP5 II on a DM 600 CFS Upright Microscope. The images were captured with the LAS AF4 software, analyzed in ImageJ2 Version 1.53c Software and presented as the maximum projection of a set of z-stacks for each stained tissue section.

### 2.9. Adult Zebrafish Heart Isolation and Histology

Adult zebrafish were euthanized in 0.016% tricaine containing 0.1 M potassium chloride to arrest the heart chambers in diastole [36]. Images of whole hearts were captured using the DFK2BUC03 camera from The Imaging Source Bremen, Germany, mounted on SMZ1000 stereoscope. Then, adult hearts were fixed in 4% paraformaldehyde at 4 °C overnight, washed three times with PBS, dehydrated in EthOH and embedded in paraffin. Paraffin sections of 5 μm thickness were performed using a Leica RM2265 microtome. Hematoxylin and eosin staining was performed according to standard laboratory protocols. Images of stained sections were captured with the Leica DFC500 camera mounted on the Leica DMLS2 microscope.

### 2.10. Estimation of Cardiac Function

High-speed videos of 30 frames per second and of 10 s duration taken under the Leica DMIRE2 microscope were used to measure and calculate heart features. Heart rate (bpm, beats per minute) was calculated by counting the number of heartbeats throughout the video and calculating the rate over 60 s. Derived from the still images of the videos, the long axis (lax) and short axis (sax) of the ventricle during end diastolic and end systolic were measured and the average of three end diastolic and three end systolic per embryo was used to calculate ventricular volumes. Assuming that the shape of the ventricle is a prolate spheroid, the EDV and ESV were calculated using the following standard formula: V = (1/6) × π × (sax)^2^ × (lax) [37]. The stroke volume was calculated by the following: SV = EDV-ESV. The cardiac output was calculated by the following: CO = Heart rate × SV. The ejection fraction was calculated by the following: EF (%) = SV/EDV × 100. In addition, the shortening fraction was calculated by the following: SF = (lax_(d)_ − lax_(s)_)/lax_(d)_.

### 2.11. Statistical Analysis

Statistical analysis and plotting were carried out in GraphPad Prism (version 5.03 for Windows). All data are presented as mean ± SD and *p*-value was considered significant * *p* ≤ 0.05, ** *p* ≤ 0.01.

## 3. Results

### 3.1. Expression Profile of Zebrafish cfdp1 Gene during Embryonic Development

Although the BCNT (Bucentaur) protein superfamily is highly conserved between species, their functional role remains unclear. We first analyzed the spatiotemporal expression of *cfdp1* during the development of zebrafish embryos, focusing on the cardiac area.

**Figure 1:**
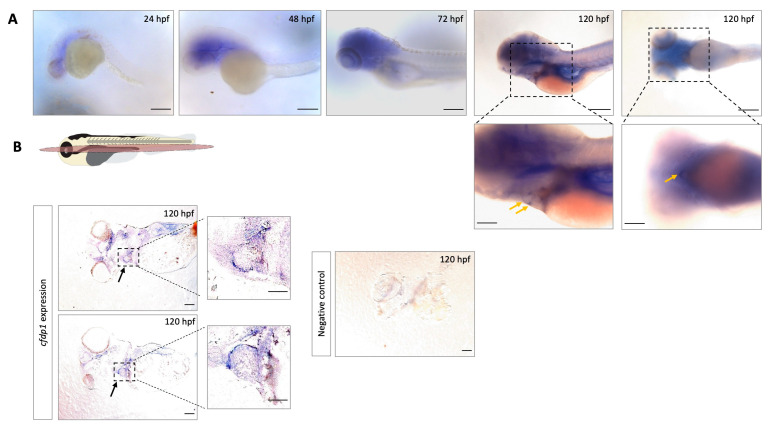
Expression analysis of *cfdp1* in different zebrafish development stages shows that *cfdp1* emerges during early development. (**A**) Whole-mount in situ hybridization of *cfdp1* in wild-type zebrafish embryos at different development stages. Higher magnification images of the lateral and ventral plane of the 120 hpf *cfdp1* ISH-stained embryos are shown in the lower panels, respectively. Arrows point at the region of the heart. The expression of the gene is apparent from 24 hpf and is restricted at the region of the head and the heart by 120 hpf. *n* = 30 in each of three independent experiments. Scale bar (upper panels) 150 μm; scale bar (lower panels) 200 μm. (**B**) Top: Illustration of frontal cutting plane of zebrafish; image panels: paraffin sections of 120 hpf ISH-stained embryos with *cfdp1* antisense RNA probe (left) and *cfdp1* sense RNA (negative control, right). Arrows point at the stained embryonic heart. Scale bar: 50 μm.

We performed whole-mount in situ hybridization (ISH) with a specific *cfdp1* antisense RNA probe in wild-type embryos. At the early developmental stages starting from 24 hpf, *cfdp1* expression was observed at the anterior part of the organism and at 120 hpf *cfdp1* was predominantly detected at the cephalic region and the developing heart (Figure 1A). Control embryos that were hybridized with the sense *cfdp1* RNA probe showed no staining pattern. In addition, earlier work has showed that maternal *cfdp1* mRNA is provided [17,18]. To further investigate the cardiac *cfdp1* expression at this later developmental stage, 120 hpf ISH-stained embryos were collected, embedded in paraffin and cut in 5 μm tissue sections via microtome. The histological analysis showed that *cfdp1* was expressed at the wall of the developing heart (Figure 1C). These data revealed for the first time *cfdp1* expression during cardiac development, suggesting that *cfdp1* might play an important role in the heart. In addition, data from the Human Heart Atlas [38] reveal that CFDP1 is also expressed in the adult heart in various tissues, including endocardial and myocardial as well as cardiac fibroblast (Supplementary Figure S1).

### 3.2. Generation of Zebrafish cfdp1 Mutant Line

**Figure 2:**
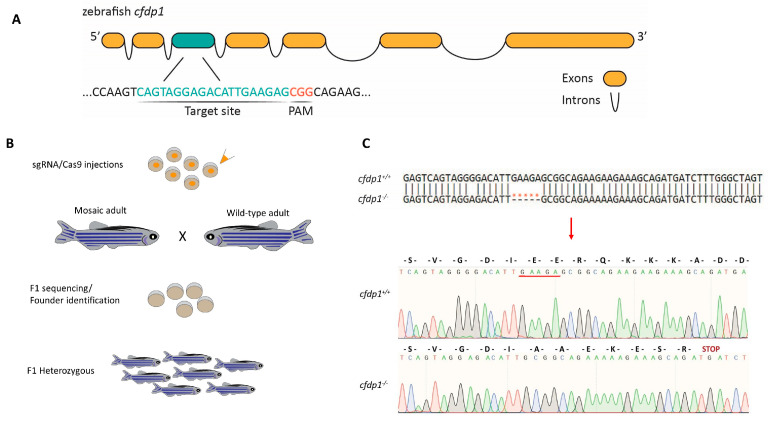
Generation of *cfdp1* zebrafish mutant line. (**A**) Schematic representation of the zebrafish *cfdp1* gene. For generation of the CRISPR/Cas9-mediated mutant line, a target site in exon 3 was selected. (**B**) Schematic representation of the strategy for CRISPR/Cas9-mediated zebrafish line. The injection mix of gRNA/Cas9 was injected at the one-cell-stage embryos. The crispants (F0-injected) grow until adulthood and are crossed with wild-type adults. The F1 generation is genotyped to identify possible founders of the line. After the identification, the corresponding F1 heterozygous generation is kept for further analysis. (**C**) Upper: nucleotide alignment between *cfdp1* mutant and *cfdp1* wild-type sequence. A 5 bp deletion in *cfdp1* mutant is detected. Lower: chromatogram of Sanger sequencing of *cfdp1* mutant and *cfdp1* wild-type sequence and the corresponding aa they encode. In the *cfdp1* mutant, at the point of DNA break, seven novel amino acids (aa) are inserted before a premature stop codon.

Following the demonstration of *cfdp1* expression in the zebrafish heart, we addressed its function via morpholino-induced knockdown and gene knockout. We used both strategies to analyze its role in the developing zebrafish heart and circumvent any phenotypic discrepancies between *cfdp1* morphants and mutants, according to previously reported cases [39]. The zebrafish *cfdp1* gene is located on chromosome 18 and consists of seven exons, which encode for a protein of 312 amino acids (aa). We utilized the online tool CHOPCHOP (https://chopchop.cbu.uib.no/) and designed a guide RNA (gRNA) to target exon 3 (Figure 2A) as it scored at the highest ranking and efficiency rating according to the published instructions [35]. The mixture of *cfdp1* gRNA and Cas9 mRNA was then injected at one-cell-stage embryos of the *Tg(myl7:EGFP)* line and the efficiency of the induced mutation was verified by the sequencing of the flanking region around the target site of the injected embryos. F0 fish were raised until adulthood when they were crossed with wild-type individuals to identify possible founders of the *cfdp1* zebrafish line and to assess whether the induced mutation was transmitted to the F1 generation (Figure 2B).

The mutated allele we selected was shown to carry a deletion of five nucleotides (NM_001100035.1:c.462_466del) causing a frameshift and leading to an introduction of a premature stop codon (p.(Glu108AlafsTer8)). More specifically, the deletion of GAAGA before the protospacer adjacent motif (PAM) sequence of the target site was predicted to result in a truncated product of 114aa, that incorporates 107aa of the wild-type *cfdp1* protein and seven new aa before harboring the premature stop codon (Figure 2C). The highly conserved BCNT domain at the C-terminal domain (exon 6 and exon 7) was also predicted to be absent in the resulting mutant product. Additionally, the deletion of the mutant allele led to the loss of the unique *SapI* cutting site in the surrounding area of the target site, which was subsequently used for genotyping the *cfdp1* siblings (Supplementary Figure S2).

### 3.3. Zebrafish cfdp1 Mutants Show Impaired Cardiac Performance

**Figure 3:**
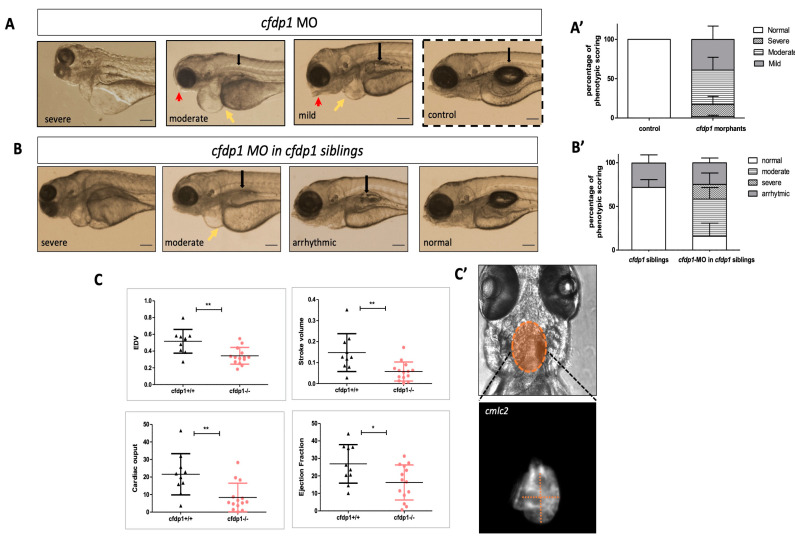
Impaired cardiac performance of *cfdp1* mutant embryonic hearts. (**A**) Stereoscopic images of representative 120 hpf *cfdp1*-MO-injected (*cfdp1* morphants) and uninjected control embryos. Black arrows point to the swim bladder, yellow arrows point to the pericardiac edema, red arrows point to the mouth opening position. Scale bar 150 μm. (**A’**) Quantification of phenotypic scoring via GraphPad Prism. Data are presented as mean ± SD. *n* = 40 in each of five independent experiments. (**B**) *cfdp1*-MO injections in sibling embryos derived from the cross between heterozygous *cfdp1* adult fish. *n* = 154 in four independent experiments. Black arrows point to the swim bladder, yellow arrows point to the pericardiac edema, red arrows point to the mouth opening position. (**B’**) Quantification of phenotype scoring of *cfdp1* siblings (pool of all three genotypes: *cfdp1^−/−^, cfdp1^−/+^*, *cfdp1^+/+^*) and *cfdp1*-MO-injected *cfdp1* sibling embryos. Data are presented as mean ± SD. Scale bar 150 μm. (**C**) Defective cardiac performance of 120 hpf *cfdp1^−/−^* embryos compared to their siblings *cfdp1^+/+^* based on ventricular measurements after recording their heart rates. Data are presented as mean ± SD, *n* = 10–14/genotype. (**C’**) Brightfield and fluorescent image of *cfdp1* mutant embryos utilizing their Tg(*myl7:EGFP*) (also referred to as *cmlc2*) background. Dashed lines indicate the long ventricular axis and the short ventricular axis, respectively.

Following the generation of the *cfdp1* mutant line, we examined the phenotype of homozygous individuals. Intriguingly, homozygous mutant larvae did not reach adulthood and survived up to 10–15 dpf. We have genotyped 59 adult fish from three different incrosses of *cfdp1^−/+^* and could not identify a homozygous mutant carrier. The ratio was 40 heterozygous *cfdp1^−/+^* and 19 *cfdp1^+/+^*. Embryos from a *cfdp1^−/+^* incross did not exhibit any gross phenotypic cardiac morphogenic malformations. However, when monitored at 5 dpf, we detected that 27.8% of them suffered from cardiac arrhythmias (N = 3, *n* = 141) (Figure 3B’ siblings, Supplementary Video S1; and compare with Supplementary Video S2). We have sequenced and genotyped over 100 embryos and larvae from multiple incrosses. When we sequenced all embryos from two incrosses, there was a Mendelian segregation of *cfdp1^−/−^*, *cfdp1^−/+^* and *cfdp1^+/+^* (16:28:13). Interestingly, when we preselected for arrhythmic fish before genotyping at 4 dpf, not only homozygous *cfdp1^−/−^* but also heterozygous *cfdp1^−/+^* siblings (approximately 10% of this arrhythmic group, *n* = 9/97, from 4 *cfdp1^−/+^* incrosses) were present with the observed heart dysfunction (Supplementary Video SM3; and compare with Supplementary Videos S4–S6). However, heterozygotes appear viable and will eventually survive, since we genotyped two-thirds of the surviving adults as *cfdp1^−/+^*. These findings highlighted the importance of *cfdp1* for proper heart function since the induced gene mutation leads to lethality, but also alludes to a partially penetrant haploinsufficiency.

In parallel, we investigated the embryonic phenotype upon silencing of *cfdp1* expression via the antisense morpholino oligonucleotide (MO)-mediated knockdown approach. We injected *cfdp1* translation-blocking MO in one-cell-stage wild-type embryos and then incubated them at 28 °C up to 120 hpf for monitoring. Figure 3A,A’ show the phenotypic scoring of *cfdp1* morphants compared to the control sibling embryos. Most of the injected embryos developed pericardial edema, from severe heart balloon shape to moderate edema, along with craniofacial malformations, defects in otoliths and body curvature. The heart malformations of the *cfdp1* morphants’ phenotype indicate a role for *cfdp1* in proper cardiac development and function. We also performed *cfdp1*-MO injections in one-cell-stage embryos from a *cfdp1^−/+^* incross. Data showed in Figure 3B that 22.3% of the injected embryos developed arrhythmic hearts; 42.1% exhibited a moderated phenotype with pericardial edema, reduced size of the head/eyes, malformations of the mouth opening and a flat or non-fully inflated swim bladder; and 7.2% were scored as a severe phenotype with gross abnormalities (N = 4, *n* = 152). Therefore, the percentage of *cfdp1* MO-injected *cfdp1* sibling embryos that developed arrhythmic hearts was slightly reduced compared to the corresponding percentage observed in *cfdp1* siblings, while the appearance of moderate and severe phenotype scoring in *cfdp1*-MO-injected *cfdp1* siblings was in accordance with the corresponding observed phenotypes in *cfdp1*-MO-injected wild-type embryos (Figure 3B,B’ and Supplementary Video S7).

We also monitored *cfdp1^−/−^* embryos and larvae and we quantified several cardiac function parameters through the high-speed video imaging of single individuals (a previously described method by Hoage et al., 2012) [37]. Following an incross of heterozygous *cfdp1^−/+^* among the embryos that developed cardiac arrhythmias, homozygous *cfdp1^−/−^* and a small percentage of heterozygous *cfdp1^−/+^* were phenotypically identical. Therefore, we recorded videos of all *cfdp1* siblings from such an incross, at 120 hpf acquiring brightfield and fluorescent images utilizing the *Tg(myl7:EGFP)* reporter line for cardiomyocytes visualization. Embryos were then sacrificed and genotyped. *cfdp1^−/−^* showed significantly reduced end-diastolic volume and stroke volume as well as cardiac output and ejection fraction, compared to wild-type *cfdp1^+/+^* siblings (Figure 3C), demonstrating the role of *cfdp1* in embryonic cardiac physiology.

These observations led us to reason that the arrhythmic phenotype is the most specific one, as there will be no mRNA in the mutants to block its translation, and to investigate if blocking of zygotic transcription, as in the case of the incross of heterozygous carriers, would lead to a more specific phenotype.

**Figure 4:**
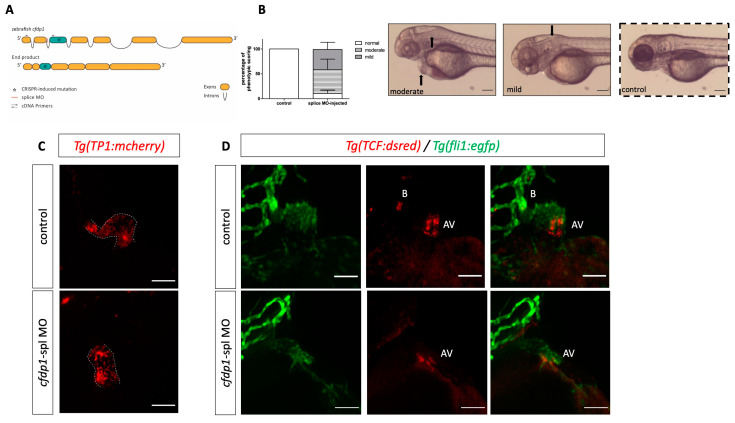
Silencing of *cfdp1* expression with a second splice-blocking morpholino. (**A**) Schematic representation of splicing outcome after microinjection of splice morpholino targeting the exon 2–intron 2 splice junction. The sequenced product shows a frameshift that results in a premature termination codon. (**B**) Stereoscopic images of representative 72 hpf *cfdp1*-splice-MO-injected and uninjected control embryos. Black arrows point to the head and heart edema and quantification of phenotypic scoring via GraphPad Prism 9. Data are presented as mean ± SD. *n* = 50 in each of three independent experiments. Scale bar 150 μm. (**C**) Max projection of fluorescent images showing that *cfdp1* silencing does not affect Notch activity at 72 hpf splice-MO-injected embryos (N = 3, *n* = 20), while (**D**) Wnt/β-catenin reporter activity is reduced compared to wild-type control siblings (N = 2, *n* = 25). AV: Atrioventricular valve; B: bulbus arteriosus; scale bar: 50 μm.

We then injected a second morpholino, targeting the second exon–intron boundary (Figure 4A). The sequencing of the *cfdp1* cDNA in morpholino-injected embryos showed that a slightly upstream cryptic donor splice site in exon 2 was used, resulting in a predicted premature stop codon at exon 3 (Supplementary Figure S3). The injected embryos exhibited smaller hearts and brain edemas but overall, a milder phenotype than the ATG-targeting morpholino (Figure 4B). Further investigation of Wnt and Notch reporter activities of 72 hpf injected embryos upon silencing via *cfdp1* splice-morpholino was in accordance with the previous *cfdp1* knockout and knockdown approaches. Notch-activated cells exhibited similar expression patterns to the wild-type controls throughout the embryonic heart (and Notch expression was restricted at the valvular regions at 120 hpf; Supplementary Figure S3B). Collectively, these findings show that *cfdp1* silencing affects cardiac development.

### 3.4. Zebrafish cfdp1 Heterozygous Develop Variation in Phenotype at Embryonic Stage

**Figure 5:**
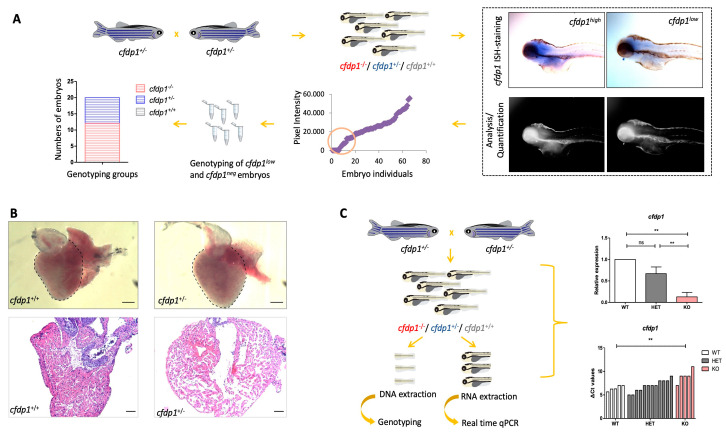
Study of *cfdp1^−/+^* embryonic and adult hearts. (**A**) Expression of *cfdp1* in *cfdp1* siblings (pool of three genotypes: *cfdp1^−/−^, cfdp1^−/+^*, *cfdp1^+/+^*). After performing in situ hybridization using the *cfdp1* RNA probe in *cfdp1* siblings (*n* = 69) at 120 hpf, ISH signal intensity was quantified. The *cfdp1*^low^ and *cfdp1*^neg^ ISH signal embryos were genotyped (*n* = 20) and it was confirmed that they corresponded to *cfdp1^−/−^* and *cfdp1^+/-^* embryos. (**B**) Brightfield images of freshly isolated whole-mount hearts (upper panels) and brightfield images of 5 μm paraffin-embedded, H&E-stained cardiac slices (lower panels) of 7 months post fertilization (mpf) *cfdp1^+/+^* (*n* = 3) and *cfdp1^+/-^* (*n* = 3) adult hearts. Scale bar (upper panels): 200 μm; scale bar (lower panels): 50 μm. (**C**) Workflow strategy. Collection of *cfdp1* siblings after cross of heterozygous *cfdp1* adult fish followed by RNA isolation and genotyping of single embryos. As shown via quantitative real-time PCR, *cfdp1* expression levels in *cfdp1^−/−^* embryos are statistically significantly reduced compared to the wild-type *cfdp1^+/+^* siblings when normalized to *act2b* as a reference gene. (**C**) *cfdp1^+/-^* embryos exhibit a range of gene expression (ΔCts plot) while a portion of heterozygous embryos present similar expression levels to the mutant embryo siblings. Student’s *t*-test (two-tailed distribution, unpaired); ** significantly different *p*-value < 0.01; ns: non-significant. Data are presented as mean ± SD.

Due to the phenotypic heterogeneity in the group of heterozygous *cfdp1^−/+^*, we initially examined whether this results from detectably different levels of *cfdp1* expression within the group. Therefore, we performed whole-mount ISH in a group of *cfdp1* siblings at 120 hpf. The images of ISH-stained single embryos were captured and the expression levels of *cfdp1* were quantified via measuring the ISH-staining pixel intensity that was analyzed using Fiji ImageJ2 software [40]. This method represents an unbiased way of quantification of the expression without prior knowledge of the genotype. After imaging, we selectively collected ISH-stained embryos that exhibited low *cfdp1* expression levels and sequenced them in order to correlate their genotype to the quantified *cfdp1* expression levels retrospectively. As expected, the heterozygous *cfdp1^−/+^* showed a broad range of *cfdp1* expression, which explains the phenotypic variation within this genotype group (Figure 5A).

Since heterozygous *cfdp1^−/+^* reach adulthood, we examined the structure of the heart of adult *cfdp1^−/+^* compared to matched-age *cfdp1^+/+^* individuals. Hence, adult fish were sacrificed, and their hearts removed, sectioned and stained with hematoxylin and eosin for nuclear and ECM/cytoplasm staining, respectively. For accurate assessment, images of all heart sections were captured and analyzed on sections revealing the cardiac valves and the largest ventricular area. We observed that heterozygous *cfdp1^−/+^* showed a dilated ventricle, with a thinner compact myocardium and sparse trabecular myocardium, with respect to wild-type siblings (Figure 5B). Overall, these findings show that the levels of cfdp1 expression could explain the respective phenotypic variability of heterozygous larvae and that *cfdp1^−/+^* adults develop defects in heart morphology.

We further analyzed the levels of nonsense-mediated mRNA decay (NMD) in individual embryos. This is a mechanism often triggered in CRISPR/Cas9-generated mutants causing a premature stop codon. Indeed, in individual *cfdp1^−/−^* embryos, *cfdp1* expression levels are statistically significantly reduced compared to the wild-type *cfdp1^+/+^* siblings (Figure 5C).

### 3.5. Abrogation of cfdp1 Expression Reduces Activation of Wnt Pathway in the Embryonic Heart but Notch Signaling Remains Unaffected

Next, based on the importance of Notch [41,42,43] and Wnt [44,45] signaling pathways for the proper development, morphogenesis and function of the embryonic heart, we investigated whether these major regulator pathways are affected by *cfdp1* abrogation. We used two well-described transgenic reporter lines as indicators for proper heart development, morphogenesis and function. Canonical Wnt/β-catenin signaling is a critical regulatory pathway for cardiac specification, differentiation and development, as the disruption of its expression results in defects in cardiac looping and endocardial cushions. Wnt and Notch have different activity patterns since Wnt activity is primarily located at the abluminal, mesenchymal-looking cells of the valves [34], while the Notch reporter line is in the endocardial cells throughout the heart and becomes restricted at the AV canal and the outflow tract [46].

**Figure 6:**
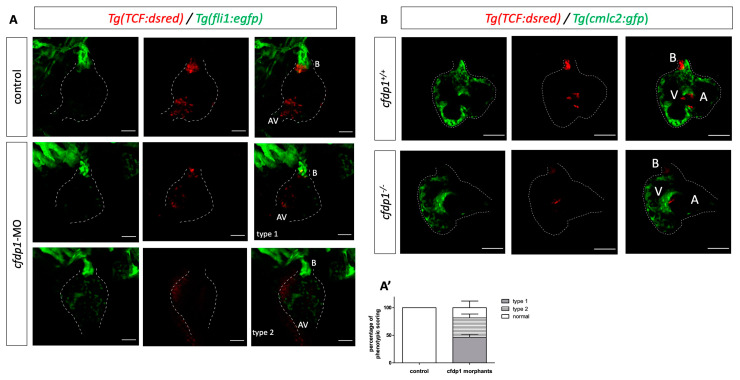
*cfdp1* abrogation shows impaired Wnt/β-catenin signaling. (**A**) Confocal images of *cfdp1* morphants show that Wnt/β-catenin reporter activity is diminished compared to the uninjected sibling controls. Max projection of z-stack confocal images of 120 hpf *cfdp1*-MO embryos. Endothelial cells are labeled with green (Tg(*fli1:EGFP*)) and Wnt-activated cells are labeled with red (Tg(7x*TCF-Xla.Siam:nlsmCherry*)); *n* = 6 in each of three independent experiments. (**A’**) percentage of phenotypic scoring. AV, atrioventricular valve. B, bulbus arteriosus. Scale bar 150 μm. (**B**) Confocal images of 120 hpf *cfdp1* mutant and wild-type siblings expressing *nlsmCherry* in Wnt-activated cells and *Tg(cmlc2:eGFP)* in all cardiomyocytes (Ν = 3, *n* = 13). Scale bar: 50 μm.

We crossed Tg(*fli1:EGFP*) (endothelial specific) with Tg(7x*TCF-Xla.Siam:nlsmCherry*) and injected the *cfdp1* morpholino. We observed a significant downregulation of Wnt activity in the *cfdp1* morphants as they appeared to develop from a moderate phenotype with a reduced Wnt reporter signal to an extreme phenotype with no fluorescence in the heart region (Figure 6A,A’).

Proceeding from the findings of the *cfdp1* knockdown approach, we investigated whether *cfdp1* mutant embryos exhibit defective Wnt activity. For this purpose, we crossed adult heterozygous *cfdp1^−/+^* carrying both Tg(*myl7:EGFP*) and Tg(7x*TCF-Xla.Siam:nlsmCherry*). The 120 hpf siblings from such an incross were screened under a fluorescent microscope and double positive egfp^+^/mCherry^+^ larvae were genotyped, while the anterior part was further processed and imaged to characterize the phenotype. As illustrated in Figure 6B, the maximum projection of z-stack imaging revealed that the Wnt pathway was significantly downregulated in the mutant *cfdp1^−/−^*embryonic hearts compared to their wild-type *cfdp1^+/+^* siblings, which was appropriately in line with the observed disruption of Wnt signaling activity in *cfdp1* morphants.

**Figure 7:**
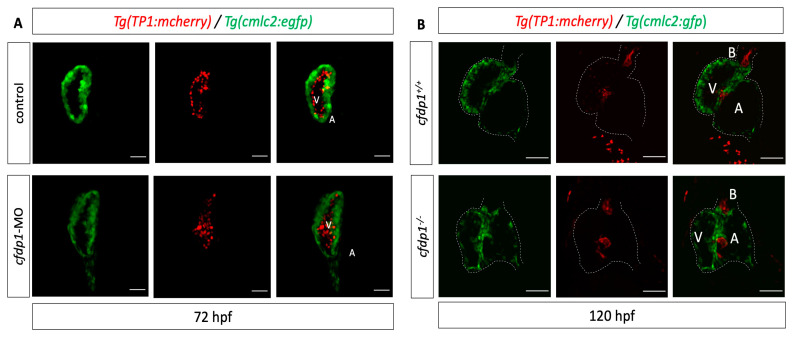
*cfdp1* abrogation does not affect Notch signaling. (**A**) Notch signaling remains unaffected in *cfdp1* morphants compared to the uninjected sibling controls. Max projection of z-stack confocal images of 72 hpf *cfdp1*-MO embryos. Ventricular cardiomyocytes are labeled with green (Tg(*myl7:GFP*)) and Notch-activated cells are labeled with red (Tg(*Tp1:mCherry*)). *n* = 10 in each of three independent experiments. Scale bar 150 μm. (**B**) Confocal images of 120 hpf *cfdp1* mutant and wild-type siblings expressing *Tg(TP1:mcherry)* in Notch-activated cells and *Tg(cmlc2:eGFP)* in all cardiomyocytes. (N = 3, n = 12) A: atrium; V: ventricle; B: bulbus arteriosus; scale bar: 50 μm.

We then utilized Tg(*myl7:GFP*) to visualize myocardial cells and Tg(*Tp1:mCherry*) which indicate the Notch-activated cells as the expression of *mCherry* is driven by the Notch-responsive element *Tp1*. The reporter line is active throughout the endocardium of the heart and then becomes restricted at the valve-forming region and more specifically at the luminal endocardial cells of immature valve leaflets [46,47]. The *cfdp1*-MO-injected embryos showed no differences in the Notch reporter activation compared to the control siblings at 72 hpf (Figure 7A). We additionally studied the effect of *cfdp1* abrogation in the Notch signaling activity of embryonic hearts, which is also involved in valve formation with respect to the corresponding findings in *cfdp1* morphants. Following a similar strategy, adult heterozygous *cfdp1^−/+^* carrying both Tg(*myl7:EGFP*)/Tg(*Tp1:mCherry*) were crossed. Remarkably, analogous to *cfdp1* morphants results, the Notch activation pattern appeared similar to the *cfdp1^−/−^* embryonic hearts compared to that of the wild-type *cfdp1^+/+^* siblings (Figure 7B). In summary, *cfdp1^−/−^* Notch-expressing endocardial cells do not exhibit any differences while TCF-positive mesenchymal-like valvular cells show lower activation levels than that of the *cfdp1^+/+^* siblings.

### 3.6. Cardiac Trabeculation in Developing Zebrafish Ventricle Is Defective in cfdp1 Mutants

**Figure 8:**
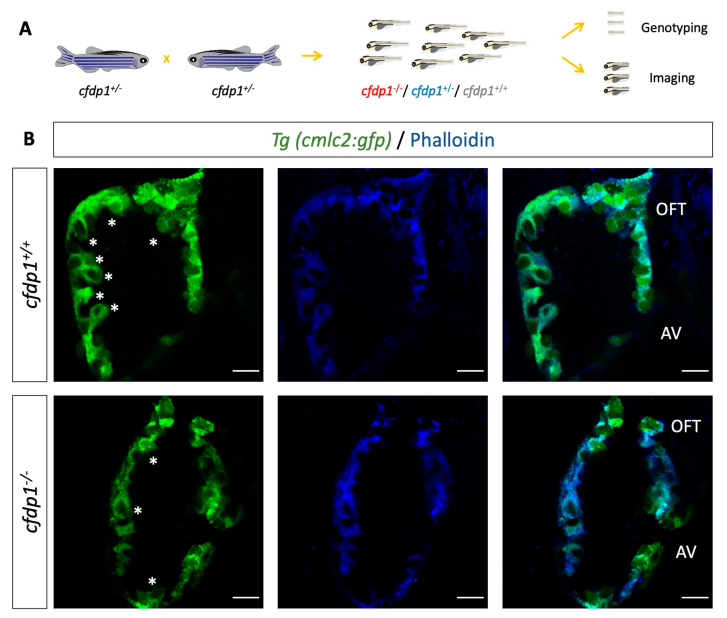
*cfdp1* is required for proper cardiac trabeculation (**A**) Schematic representation of retrospective analysis. (**B**) Single confocal plane of fluorescent phalloidin staining (actin filaments) in 120 hpf *cfdp1* embryos, expressing *Tg(cmlc2:eGFP)* in all cardiomyocytes. Asterisks: trabeculae cardiomyocytes; AV: atrioventricular; OFT: outflow tract. *n* = 5 in each of three independent experiments Scale bar: 50 μm.

Prior studies have shown that the orchestration of cardiac trabeculation is highly significant for proper heart function and embryo survival. Defects in trabeculation lead to embryonic lethality or adult dilated cardiomyopathies [48,49]. It has also been shown that zebrafish *erbb2* mutant embryos lack trabeculation but they develop normal valves [50]. To this end, we examined the levels of cardiac trabeculation in *cfdp1* mutant embryos at 120 hpf when the entire length of the luminal side of the ventricle has developed extensive trabeculation. *Cfdp1* embryos were genotyped at 120 hpf and wild-type and mutant embryos were stained with phalloidin for filamentous actin staining (Figure 8A). Interestingly, while wild-type *cfdp1* sibling embryos developed a normal pattern of trabeculation, *cfdp1^−/−^* embryos exhibited a less complex trabeculation network as the emergence of delaminated endocardial cells in the ventricular lumen of the heart was diminished (Figure 8B). This finding suggests the requirement of *cfdp1* for the proper initiation and formation of the trabecular cardiomyocyte layer. However, since trabeculation depends on proper intracardiac blood flow dynamics [50], we cannot rule out the possibility that the effect is secondary to the impaired cardiac function we measured in *cfdp1^−/−^* larvae at earlier stages.

## 4. Discussion

### 4.1. A Model for the Phenotypic and Functional Characterization of cfdp1

Genome-wide association studies have unraveled multiple genome loci associated with human diseases. A recent study performed deep transcriptomic analysis of genotyped primary human coronary artery smooth muscle cells (HCASMCs) and coronary endothelial cells (HCAECs) from the same subjects and analyzed GWAS loci associated with vascular disease and CAD risk in these two coronary cell types [51]. Researchers found *CFDP1* (along with *YAP1* and *STAT6*) for HCAECs that passed the 5% false discovery level (FDR) correction at the gene level which associates *CFDP1* with artery disease traits [51]. Another study which applied a two-stage discovery and replication study design with more than 15,000 individuals identified the association of a novel SNP in the last 3′ intron of *CFDP1*, rs4888378, with carotid intima–media thickness (cIMT), an established marker for subclinical atherosclerotic cardiovascular disease [9]. A different study identified another *CFDP1* variant, rs3851738, as a CAD-associated locus after analysis from the UK Biobank and CARDIoGRAMplusC4D 1000 Genomes imputation study, and following ‘phenome-wide association study’ (PheWas) correlated this variant with systolic blood pressure [52]. In the same context, GWAS studies have shown the correlation of human *CFDP1* with aortic root diameter, as well as CAD risk [6,53]. A recent GWAS study focusing on the characterization of risk variants for CAD incorporated the Polygenic Priority Score (PoPS) to enhance the prioritization of causal genes within a CAD-association locus [25]. The genes *BCAR1*, *CFDP1* and *TMEM170A* were implicated in CAD potential pathogenesis whilst *CFDP1* was specifically related to the regulation of cell shape. Of note, although *CFDP1* was the nearest gene to the CAD-associated lead variant rs8046696, *BCAR1* was suggested as the prioritized gene of this locus based on the concordance of CAD predictors and the integration of reported phenotype data from the Mouse Genome Informatics database (http://www.informatics.jax.org/ accessed on 1 March 2023). The examination of the lead SNP rs4888378 (intronic in *CFDP1*) at the *BCAR1-CFDP1-TMEM170A* locus suggested the distal interaction of rs4888378 and implicated *BCAR1* as a causal variant [8]. Interestingly, BCAR1 levels were also significantly suppressed in the *Bcnt1/Cfdp1* knockout mouse embryonic cell line [24]. The synteny of the locus is conserved in zebrafish and, therefore, it is crucial to acutely analyze the relevant phenotypes of the genes that belong to this specific synteny in order to assess the prospective clinical significance of the CAD locus.

### 4.2. cfdp1 Knockdown and Knockout Zebrafish Demonstrated Similar but Not Identical Phenotypes

The targeted knockdown of genes via MO injections is distinguishable from stable genetic lines which inherit the induced change, since Mos are gradually degraded within a few days and therefore result in a transient effect. Despite that fact, the knockdown approach in zebrafish remains an in vivo phenotypic assay to investigate the effect of gene silencing. Our data showed that *cfdp1* morphants develop phenotypic abnormalities, such as pericardial edema, craniofacial malformations and hypoplastic swim bladder (the arrest of swim bladder inflation has been proposed to be a secondary event to heart failure, since in *silent heart* morphants that lack heart contractility, heart-specific constitutively activated AHR signaling and TCDD-exposed zebrafish models which develop heart failure, the swim bladder development is inhibited in the same manner [54]). Interestingly, the *cfdp1* mutants exhibit more mild phenotypic characterization by developing arrhythmic embryonic hearts but not pericardial edema or extreme craniofacial disorders compared to *cfdp1* morphants at 120 hpf. At the same context, when we investigated the effect of *cfdp1* depletion in the Wnt signaling pathway at *cfdp1* morphant hearts, we observed a major reduction in signal intensity or even complete blockage of the expression pattern in Wnt-activated cells of the Tg(7x*TCF-Xla.Siam:nlsmCherry*) reporter line, while *cfdp1* mutant hearts showed a strong inhibitory effect without the total silence of the Wnt pathway. It is important to note that in this manuscript the Notch and Wnt reporter lines are merely used as cell type markers, very well described in the context of heart development, and it is still unclear if *cfdp1* directly regulates either of these pathways. In addition, the differences in the manifestation of the phenotypes between knockout and knockdown embryos could possibly be justified by the activation of a genetic compensation response, which has been previously proposed to explain phenotypic discrepancies in morphants and mutant models [39].

The generation of a stable *cfdp1* zebrafish mutant line resulted from the introduction of a premature termination codon (PTC) in *cfdp1*. The detailed phenotypic study of embryos showed that *cfdp1^−/−^* embryos have arrhythmic hearts. Notably, the same phenotype was observed in a percentage of heterozygous *cfdp1^−/+^* embryos. We further investigated whether this could be a result of a variation in *cfdp1* expression levels and, indeed, we detected differences in signal intensity within the *cfdp1^−/+^* embryo pool, suggesting that this could modulate the phenotypic variation of heterozygous zebrafish. Our data support the existence of heterogeneity (variation of phenotype) in heterozygous *cfdp1* embryos and the possible correlation of wild-type/mutated copies and phenotypic outcome. A proposed scenario for this variation rests on the activation of quality-control nonsense-mediated mRNA decay (NMD) that targets flawed messenger RNAs. Since our *cfdp1* mutation induces a PTC that is not at the last exon and is ~50 nucleotides upstream of the last exon–exon junction, it is well assumed that this triggers NMD machinery [55], a surveillance pathway that degrades transcripts containing PTCs in order to maintain transcriptome homeostasis [56,57]. Although NMD plays a beneficial role by limiting the dominant-negative effect of mutant proteins, there is a variation in the efficiency of NMD activity in cell-, tissue- and transcript-specific differences that modulates the manifestation of a disorder [56,57]. NMD variation potentially leads to different clinical outcomes in individuals carrying the same PTC-containing mutated transcript [58]. Thus, the efficacy of NMD could vary between individuals and could act as a potential modifier of the disease phenotype. Therefore, the observed variability between *cfdp1^−/+^* individuals could also be a consequence of incomplete NMD, resulting in the *cfdp1* haploinsufficiency and heterogeneity observed in heterozygous carriers.

### 4.3. The Role of cfdp1 in Ventricular Trabeculation and Cardiac Function

We have shown that *cfdp1* zebrafish mutants suffer from an impaired trabecular network. Defects of this complex cardiac remodeling lead to embryonic lethality, which illustrates the importance of this process and the need to fully unravel the signaling molecules regulating the trabeculation in cardiac development. Mechanical forces and contractility are also important factors for proper trabeculation network formation. Both the reduction of blood flow in *weak atrium* (*myh6*) [50,59,60] mutants and the disrupted contractility in *silent heart* (*tnnt2a)* [60] mutants result in severe defects in trabeculation, as well as in *tnnt2a* morphants that do extend ventricular protrusions but they are less stable and frequently retract [61]. Disorder in the trabeculae layer shown in *cfdp1* mutant embryos could be a secondary event of reduced contractility which is demonstrated by reduced stroke volume and ejection fraction cardiac performances. Interestingly, zebrafish *tomo*-seq genome-wide transcriptional profiling [62] reveals a similar expression pattern of *cfdp1* (previously misassigned as *rltpr*) and *nrg1* in regenerating the heart three days after injury. Zebrafish *erbb2* mutant embryos lack cardiac trabeculation and develop progressive cardiac dysfunction and fatal heart failure, showing the functionally conserved role of Nrg/ErbB signaling in heart morphogenesis [49]. Notably, *nrg2a^−/−^* are recognized morphologically by their aberrant jaw and swim bladder inflation disorders, reminiscent of the phenotypic characterization of *cfdp1* morphant embryos. Whether the *cfdp1* mechanism of action and its role in trabeculae cardiomyocytes regulation crosslinks with the Nrg signaling pathway remains to be clarified.

The cardiac conduction system is composed of pacemaker cells in the sinoatrial junction, atrioventricular node and ventricular conduction system, and the canonical Wnt pathway has been implied to contribute during specific stages of conduction [63]. Canonical Wnt5b signaling has been reported to play an important role in heart contractility by promoting the pacemaker cardiomyocyte differentiation transcription factors Isl1 and Tbx18 and inhibiting Nkx2.5, both in zebrafish and in human pluripotent stem cells (hPSCs) [64]. Likewise, Wnt signaling activation (via Wnt3 ligand) promotes pacemaker lineage in mouse and human embryonic stem cells [65]. In *Isl1*-deficient zebrafish and mouse embryos, there is a progressive failure of contractility leading to arrhythmias and bradycardia [66] and it is reported that canonical Wnt/β-catenin signaling in zebrafish is activated in *isl1^+^* cells in the sinoatrial region affecting the control of the heart rate [67]. In addition, Wnt/β-catenin signaling in the AV canal regulates specific electrophysiological properties of the AVC and AV node by slowing down conduction velocity [68]. It has been recently reported that CFDP1 is also a neuroblastoma (NB) susceptibility gene [10]. The authors show in this manuscript that it regulates several noradrenergic neuronal genes that could explain the arrythmias in our zebrafish model. We reported that *cfdp1* embryonic mutant hearts exhibit arrhythmias, a phenotype indicating defects in contractility and pacemaker activity. Having highlighted the significant role of Wnt in regulating pacemaker development in zebrafish, *cfdp1* is suggested to function in the regulatory mechanism upstream of the Wnt pathway involved in the cellular specification of conductivity. The mechanism of how *cfdp1* cooperates with canonical Wnt/β-catenin signaling remains to be elucidated.

## 5. Conclusions

In this study, we describe for the first time the essential role of *cfdp1* in cardiac development and function. We generated a CRISPR/Cas9-induced zebrafish *cfdp1* mutant line by targeting the third exon of *cfdp1*. A deletion of five nucleotides results in the alteration of the open reading frame and consequently the introduction of seven novel amino acids followed by a premature stop codon. The predicted truncated protein product lacks the evolutionary conserved BCNT domain and the *cfdp1^−/−^* embryos die at approximately 10–16 dpf. Our work demonstrated the cardiac dysfunction upon *cfdp1* abrogation which was reflected in impaired heart features and was manifested by decreased end-diastolic volume, stroke volume, cardiac output, ejection fraction and ventricular trabeculation. The *cfdp1* mutant embryos exhibited impaired contractility, bradycardia and arrhythmias, which were also detected in some *cfdp1^−/+^* heterozygous embryos. In addition, we showed that Wnt signaling is downregulated in the mesenchymal valvular cells of *cfdp1* mutant hearts while Notch activation in the atrioventricular boundary and the initiation of valve formation remain unaffected. While Notch restriction in the AV and outflow tract indicates successful dedifferentiation of these regions, the defect in Wnt signaling indicates valve development deficiency. Both effects on valve development and differentiation as well as trabeculation are dependent on cardiac function and intracardiac flow dynamics [50,59]. Therefore, the cell-autonomous role of Bcnt/Cfdp1 in both of these processes requires further validation. We are currently looking into the cardiac innervation of homozygous larvae and heterozygous larvae and adults using acetylated tubulin among other markers. We are also looking at coronary artery patterning using the Tg(*fli1*:EGFP) transgenic line. It is possible that the common origin of neural crest cell identity for coronary arteries, sympathetic ganglia and adrenal medulla might underlie the role of CFDP1 in both NB and CAD, as well as in different congenital heart diseases.

## Data Availability

Not applicable.

## Supplementary Materials

The following supporting information can be downloaded at: https://www.mdpi.com/article/10.3390/cells12151994/s1, Supplementary Figures: *SF1*: Visualization of *cfdp1* expression analysis in major cardiac cell types in adult hearts based on Human Cell Atlas platform (https://www.heartcellatlas.org/ accessed on 1 February 2022). *SF2*: Diagnostic test after digestion of DNA samples with *SapI* restriction enzyme. KO, knockout; Het, heterozygous; WT, wild-type. Asterisk: DNA fragment after digestion; triangle: undigested DNA fragment. *SF3*: Silencing of *cfdp1* expression via splice morpholino microinjections. (A) Schematic representation of splicing outcome after microinjection of splice morpholino targeting the exon 2–intron 2 splice junction. (B) cDNA expression analysis of *cfdp1* morphants compared to uninjected control embryos via RT-PCR at 30 hpf. An upstream cryptic splice donor is used and the predicted product carries a frameshift sequence that results in a premature termination codon. (C) Fluorescent images of splice-MO-injected 120 hpf embryos showing similar levels of AV restriction of *Notch1* activity compared to wild-type control embryos; scale bar 50 μm. Supplementary Movies: *S1:* Brightfield video capture of a 5 dpf *cfdp1*^+/+^ larva. *S2:* Brightfield video capture of a 5 dpf *cfdp1*^−/−^ larva. *S3:* Fluorescent video capture [*Tg(cmlc2:eGFP)*] of a 5 dpf *cfdp1*^+/+^ larva. *S4:* Fluorescent video capture [*Tg(cmlc2:eGFP)*] of a 5 dpf *cfdp1*^−/−^ larva. *S5:* Fluorescent video capture [*Tg(cmlc2:eGFP)*] of a 5 dpf *cfdp1*^+/−^ sibling larva with normal heart rate. *S6:* Fluorescent video capture [*Tg(cmlc2:eGFP)*] of a 5 dpf *cfdp1*^+/−^ sibling larva with arrhythmic heart. *S7:* 3 dpf *cfdp1*-MO injected embryo.
