# Supplementary figures and images for: Cfdp1 Is Essential for Cardiac Development and Function
Source: Cells. 2023 Aug 3;12(15):1994. doi: 10.3390/cells12151994 (PMC10417793; doi:10.3390/cells12151994)

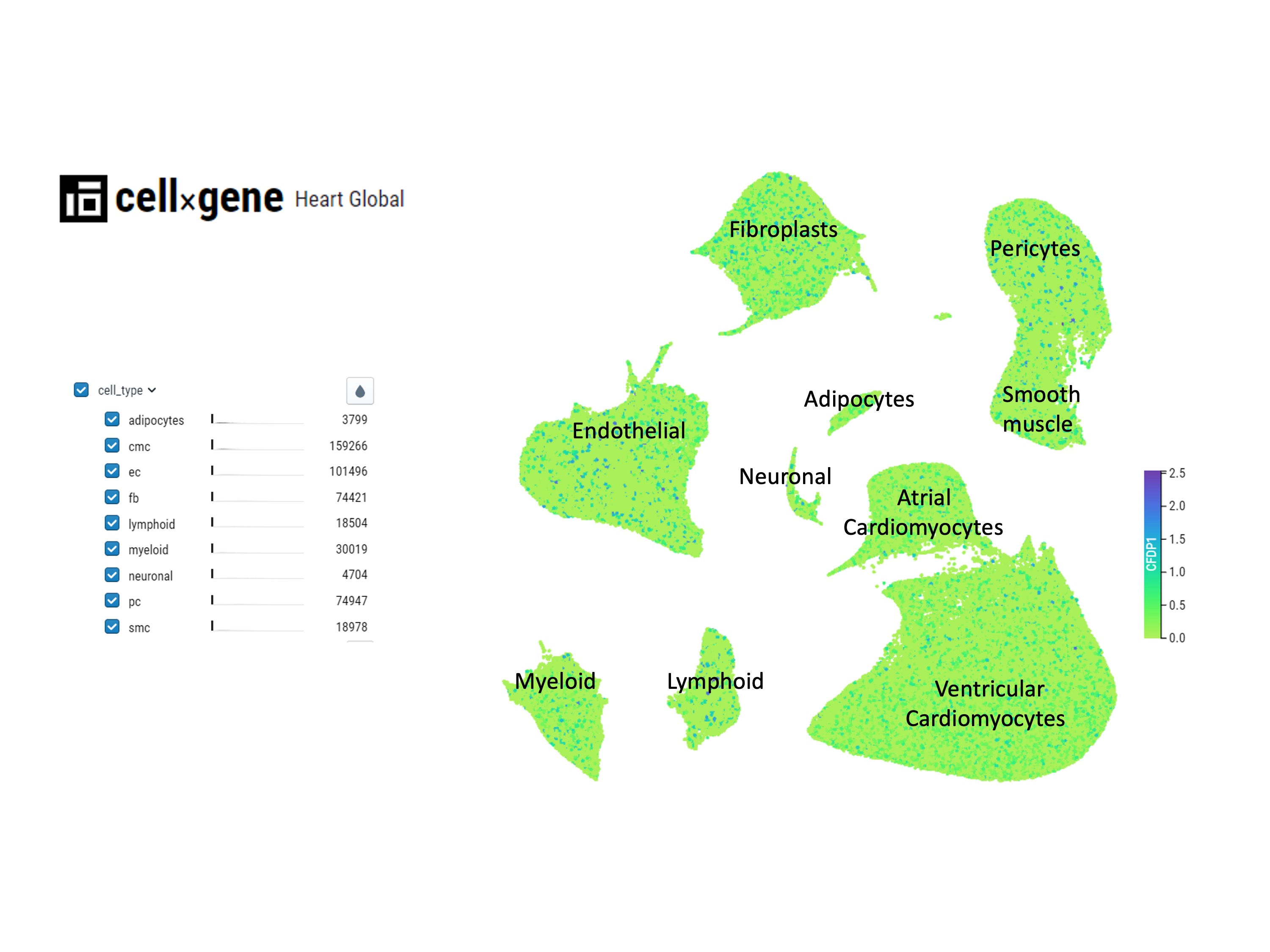

Supplement: Supplementary file 1 [file cells-12-01994-s001.zip › Suppl Figure S1.jpeg]

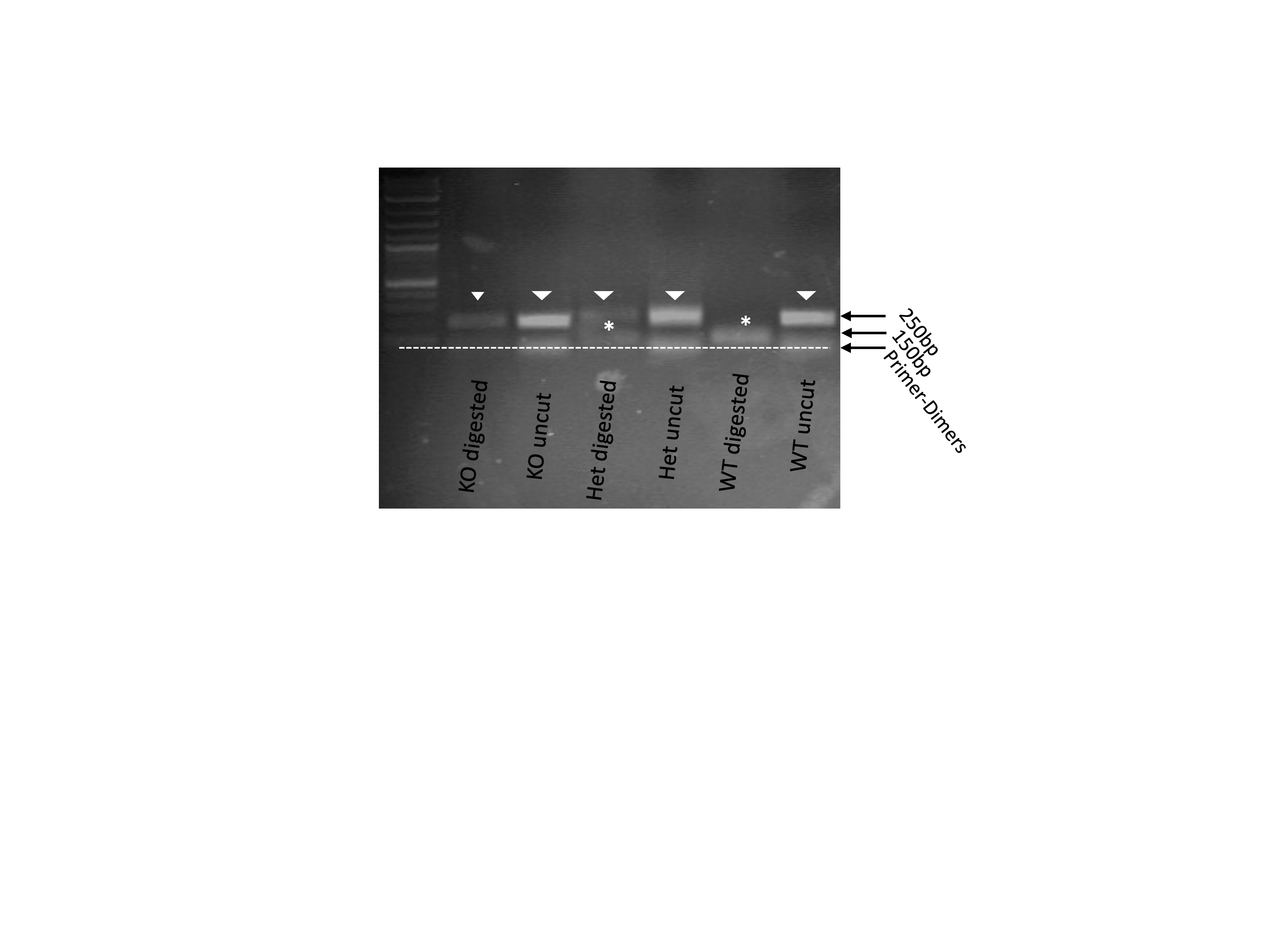

Supplement: Supplementary file 1 [file cells-12-01994-s001.zip › Suppl Figure S2.jpeg]

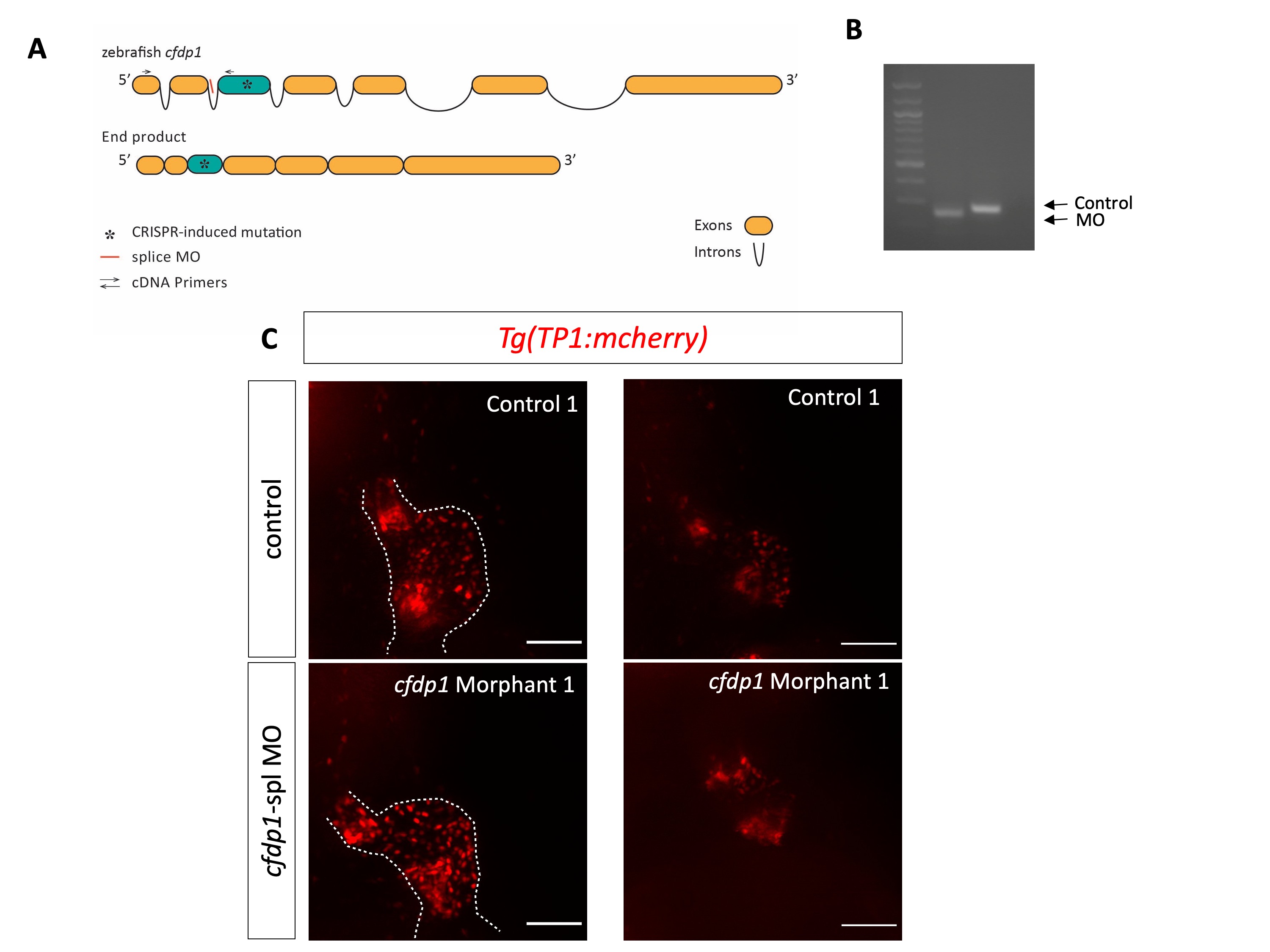

Supplement: Supplementary file 1 [file cells-12-01994-s001.zip › Suppl Figure S3.jpeg]
